# Fluoropyrimidines: A Cardiotoxicity Case for Caution

**DOI:** 10.1016/j.jaccas.2025.104738

**Published:** 2025-08-27

**Authors:** Philipp von Stein, Jonas Wörmann, Roman Pfister

**Affiliations:** Faculty of Medicine and University Hospital Cologne, Clinic III for Internal Medicine, University of Cologne, Cologne, Germany

**Keywords:** 5-FU, cardiac adverse effects, coronary vasospasm, fluoropyrimidine

## Abstract

**Background:**

Fluoropyrimidines are widely used in cancer treatment but can cause cardiac adverse effects.

**Case Summary:**

A 57-year-old man with esophageal cancer, receiving fluoropyrimidine-based therapy, was referred for preoperative cardiac evaluation. Abnormal electrocardiogram findings raised concern for ischemia, but coronary angiography ruled out obstructive disease. Fluoropyrimidine-induced coronary vasospasm was suspected, and calcium channel blocker therapy was initiated. Five months later, after tumor recurrence, continuous fluoropyrimidine infusion was restarted without cardiology consultation, and calcium channel blocker therapy had been discontinued. The patient developed cardiogenic shock and died.

**Why Beyond the Guidelines:**

Current guidelines provide limited recommendations for managing suspected fluoropyrimidine-induced coronary vasospasm.

**Discussion:**

Fluoropyrimidine-induced coronary vasospasm poses a clinical challenge. Retreatment may be feasible if obstructive coronary artery disease is excluded, and prophylactic vasospasm therapy is administered within a structured protocol.

**Take-Home Messages:**

Fluoropyrimidines can cause life-threatening coronary vasospasm. With multidisciplinary care, continued therapy may be possible. Awareness among oncologists, cardiologists, primary care physicians, and patients is critical.

## Graphical Abstract

**Figure:**
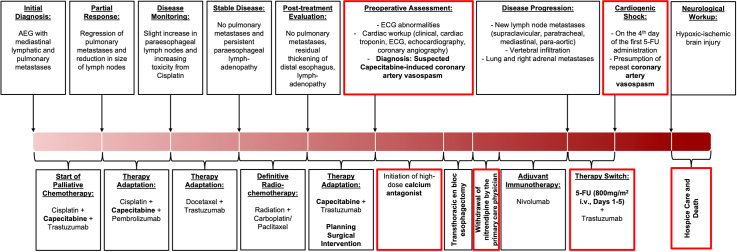

Fluoropyrimidines, including 5-fluorouracil (5-FU) and its oral prodrug capecitabine, are cornerstone agents in the treatment of various gastrointestinal and other solid tumors. Despite their efficacy, these agents are associated with potentially severe cardiac toxicities.^1^ This case highlights the need for heightened clinical vigilance and a structured, multidisciplinary approach to managing cardiotoxicity in patients undergoing fluoropyrimidine-based chemotherapy.

## Case Summary

A 57-year-old man was referred to our cardio-oncology clinic by the visceral surgery team for preoperative cardiac evaluation before planned esophagectomy. His medical history included arterial hypertension, type 2 diabetes mellitus, former tobacco use, and adenocarcinoma of the esophagogastric junction. He had previously received treatment with cisplatin, capecitabine, pembrolizumab/trastuzumab, and neoadjuvant chemoradiotherapy. His regular medications included pantoprazole, sitagliptin, capecitabine, and metamizole as needed.

**Figure 1:**
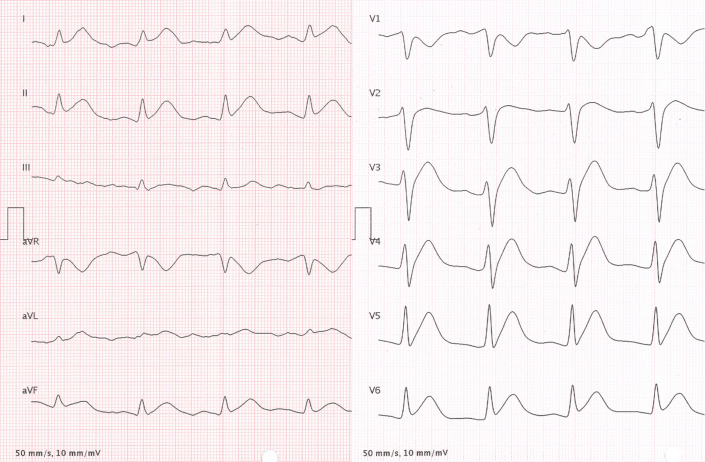
12-Lead ECG at Presentation to the Visceral Surgery Department 12-lead ECG (10 mm/mV, 50 mm/s) at presentation to the visceral surgery department at 8 am, presenting hyperacute T waves in leads I, II, aVF, and V_3_ to V_6_. ECG = electrocardiogram.

**Figure 2:**
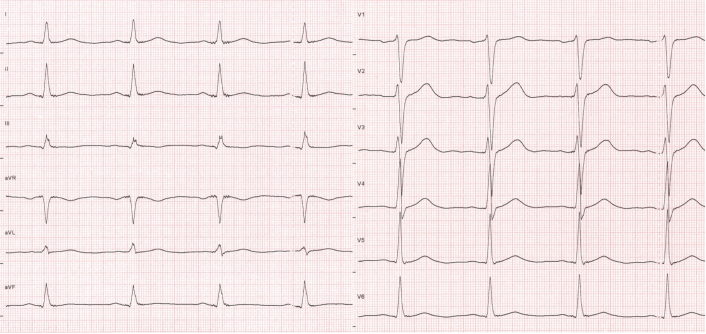
12-Lead ECG at Presentation to the Cardiology Outpatient Clinic 12-lead ECG (10 mm/mV, 50 mm/s) at presentation to our cardiology outpatient clinic at 10 am with resolution of ST-segment elevations. ECG = electrocardiogram.

The patient reported intermittent, stabbing chest discomfort radiating from the tumor region, beginning around the time of diagnosis and capecitabine initiation, and self-managed with metamizole. This discomfort also occurred during his preoperative assessment. A 12-lead electrocardiogram (ECG) obtained by the visceral surgery team showed hyperacute T waves in leads I, II, aVF, and V_3_ to V_6_ (Figure 1), raising concern for myocardial ischemia. On presentation to our outpatient clinic, he was asymptomatic, and a repeat ECG showed unremarkable findings (Figure 2). Initial high-sensitivity troponin T was 0.012 μg/L (reference <0.014 μg/L), rising to 0.021 μg/L one hour later. N-terminal pro–B-type natriuretic peptide was within normal limits (94 ng/L; reference <386 ng/L). Transthoracic echocardiography demonstrated normal left ventricular systolic function without regional wall motion abnormalities (Videos 1 to 4).

Invasive coronary angiography revealed mild-to-moderate stenosis in the right coronary artery, with no indication for revascularization (Videos 5 to 11). No coronary spasm provocation testing was performed. Given the clinical presentation, dynamic ECG changes, modest troponin rise, and ongoing capecitabine exposure, fluoropyrimidine-induced coronary vasospasm was suspected. High-dose calcium channel blocker therapy was initiated, and low-dose aspirin and statin therapy were prescribed for secondary prevention. Further fluoropyrimidine therapy was discouraged unless administered under a structured retreatment protocol with cardiology involvement.

## Why Beyond the Guidelines

Fluoropyrimidine-induced coronary vasospasm presents a therapeutic challenge, particularly as these agents remain indispensable in the treatment of various malignancies. However, current cardio-oncology guidelines offer limited guidance for managing suspected cardiotoxicity, especially regarding retreatment strategies after an initial adverse event.^2^

## Case Outcome and Follow-Up

Five months after esophagectomy, the patient had recovered well. Despite adjuvant treatment with the checkpoint inhibitor nivolumab, tumor progression was observed. The local oncology team initiated systemic therapy with trastuzumab and continuous intravenous 5-FU at a dose of 800 mg/m^2^ per day, administered over 5 consecutive days every 3 weeks. Although the patient had a documented history of fluoropyrimidine-associated coronary vasospasm, no cardiology consultation was obtained, and no adaptation of the treatment protocol was made. Notably, the previously prescribed calcium channel blocker therapy had been discontinued several months earlier by the patient's primary care physician for unknown reasons.

**Figure 3:**
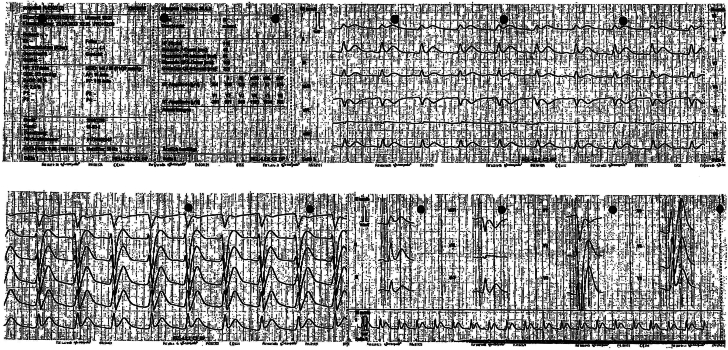
Postresuscitation ECG 12-lead ECG obtained shortly after resuscitation for ventricular fibrillation on day 4 of the first 5-FU cycle, showing hyperacute T waves in leads II and V_2_ to V_6_. 5-FU = 5-fluorouracil; ECG = electrocardiogram.

**Figure 4:**
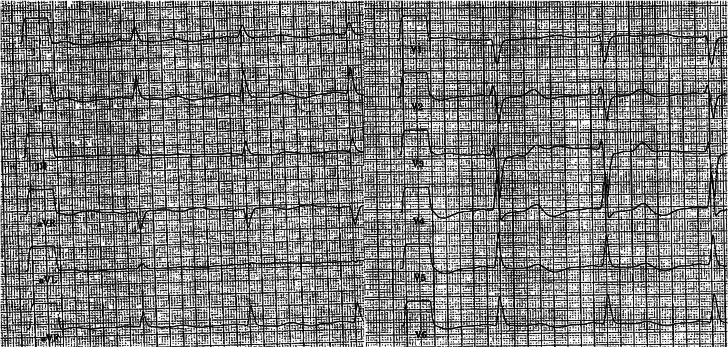
12-Lead ECG Obtained at Hospital Admission Following Resuscitation 12-lead ECG (10 mm/mV, 50 mm/s) at hospital admission showing slight ST-segment depressions in the precordial leads. ECG = electrocardiogram.

On day 4 of the first 5-FU cycle, the patient developed cardiogenic shock. Following an undocumented no-flow time, resuscitation was initiated, and ventricular fibrillation was terminated after 14 defibrillation attempts. Hyperacute T waves in leads II and V_2_ to V_6_ were evident, as shown in postresuscitation ECGs, obtained by emergency medical services, depicted in Figure 3. The admission ECG showed slight ST-segment depression in the precordial leads (Figure 4). Repeat coronary angiography showed no progression of coronary artery disease and no evidence of occlusion. Repeat transthoracic echocardiography revealed diffuse hypokinesia with a moderately reduced left ventricular ejection fraction of approximately 40%. Recurrent fluoropyrimidine-induced coronary vasospasm was presumed. The patient sustained hypoxic-ischemic brain injury and was transferred to hospice care, where he died 14 days later.

## Discussion

Fluoropyrimidines, including 5-FU and its oral prodrug capecitabine, are essential in the treatment of multiple solid tumors.^1^ However, their use is associated with a spectrum of cardiac toxicities, including coronary vasospasm, myocardial infarction, arrhythmias, cardiomyopathy, and sudden cardiac death, with reported incidences ranging from 1% to 30%.^1^

Although the precise mechanisms remain incompletely understood, fluoropyrimidine-induced cardiotoxicity is a clinically relevant, under-recognized, and insufficiently acknowledged adverse event, as exemplified by the case presented here. Conflicting reports exist regarding the predisposition of 5-FU–associated cardiotoxicity through established cardiovascular risk factors.^3^ Nonetheless, identifying patients at high risk for 5-FU cardiotoxicity could facilitate strategies for risk reduction before chemotherapy.^3^ Currently, educating patients about cardiac toxicity symptoms before treatment, raising awareness, and increasing vigilance regarding 12-lead ECG signs and clinical symptoms—such as typical or atypical chest pain—for 5-FU cardiotoxicity are essential to prevent such serious outcomes.

Given the patient's prior treatment with immune checkpoint inhibitors, including pembrolizumab and nivolumab, and the presence of impaired left ventricular function, immune checkpoint inhibitor–associated myocarditis could have been considered as part of the differential diagnosis. However, the clinical presentation and electrocardiographic findings, occurring in close temporal relation to 5-FU re-exposure, favored recurrent fluoropyrimidine-induced coronary vasospasm as the most likely cause. Nonetheless, maintaining a broad differential when assessing cardiac events in patients receiving multiple oncologic therapies remains essential.

Withholding potentially life-saving cancer therapy with 5-FU from all patients with symptoms of cardiotoxicity is not unconditionally required. Thus, Padegimas and Carver^4^ have proposed a diagnostic and therapeutic algorithm for the continuation of fluoropyrimidine therapy with concomitant administration of antianginal agents, which could offer patients with symptoms of cardiotoxicity the opportunity to continue fluoropyrimidine therapy.

## Conclusions

This case illustrates the potentially fatal cardiac complications associated with fluoropyrimidine therapy and emphasizes the critical importance of educating both physicians and patients to promptly recognize symptoms of cardiac toxicity. Treatment discontinuation is not always required; instead, a carefully monitored retreatment with prophylactic antianginal therapy may be a viable option. Multidisciplinary collaboration is essential to effectively balance oncological efficacy with the management of life-threatening cardiotoxicity.

**Visual Summary:**
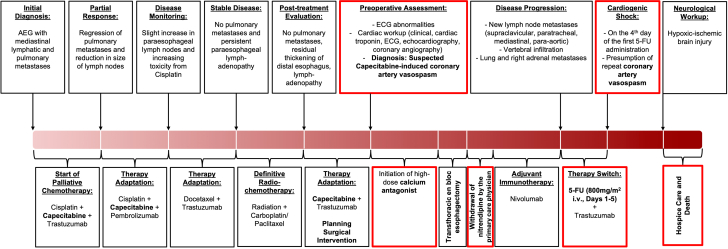
Overview of the Diagnostic and Therapeutic Course 5-FU = 5-fluorouracil; AEG = adenocarcinoma of the esophagogastric junction; ECG = electrocardiogram; i.v. = intravenous.

## Funding Support and Author Disclosures

The authors have reported that they have no relationships relevant to the contents of this paper to disclose.

**Take-Home Messages**

• Fluoropyrimidines can cause life-threatening cardiac adverse events, including coronary artery spasm, myocardial infarction, and sudden cardiac death.
• This case highlights the importance of patient and physician awareness of chemotherapy-related cardiotoxicity symptoms for timely recognition and multidisciplinary management.

## Appendix

Video 1 Apical 4-Chamber View on Transthoracic Echocardiography Transthoracic echocardiographic apical 4-chamber view demonstrating normal global left ventricular systolic function without regional wall motion abnormalities.

Video 2 Apical 2-Chamber View on Transthoracic Echocardiography Transthoracic echocardiographic apical 2-chamber view demonstrating normal global left ventricular systolic function without regional wall motion abnormalities.

Video 3 Apical 3-Chamber View on Transthoracic Echocardiography Transthoracic echocardiographic apical 3-chamber view demonstrating normal global left ventricular systolic function without regional wall motion abnormalities.

Video 4 Parasternal Short-Axis View on Transthoracic Echocardiography Short-axis view at the midventricular level confirming homogeneous myocardial contraction without regional wall motion abnormalities.

Video 5 Coronary Angiography in Left Anterior Oblique View of the Right Coronary Artery Selective coronary angiography in left anterior oblique projection demonstrating a mild-to-moderate stenosis in the midsegment of the right coronary artery.

Video 6 Coronary Angiography in Left Anterior Oblique/Cranial View of the Right Coronary Artery Selective coronary angiography in left anterior oblique/cranial projection demonstrating a mild-to-moderate stenosis in the midsegment of the right coronary artery.

Video 7 Coronary Angiography in Caudal View of the Left Coronary Artery Selective coronary angiography in caudal projection demonstrating unobstructed left anterior descending and left circumflex arteries.

Video 8 Coronary Angiography in Left Anterior Oblique/Cranial View of the Left Coronary Artery Selective coronary angiography in left anterior oblique/cranial projection demonstrating an unobstructed left anterior descending artery.

Video 9 Coronary Angiography in Right Anterior Oblique View of the Left Coronary Artery Selective coronary angiography in right anterior oblique projection demonstrating an unobstructed left anterior descending artery.

Video 10 Coronary Angiography in Left Anterior Oblique/Caudal View of the Left Coronary Artery Selective coronary angiography in left anterior oblique/caudal projection demonstrating an unobstructed left anterior descending artery.

Video 11 Coronary Angiography in Left Anterior Oblique/Caudal View of the Left Coronary Artery Selective coronary angiography in left anterior oblique/caudal projection demonstrating an unobstructed left circumflex artery.
